# 

**Published:** 1911-06

**Authors:** 


					﻿PUBLISHED MONTHLY.	ATLANT \	SUBSCRIPTION $1.00
JOUP\ \I-R! COUI)
OF medio^jEX^
(Atlanta Medical and Surgical Journal, Established 1855.
I and Southern Medical Record, Established 1870.	1 Julll lUul
Vol. LVII.	Atlanta, Ga., June, 1911.	No. 3
				

